# Evolution of anxiety management in prostate biopsy under local anesthesia: a narrative review

**DOI:** 10.1007/s00345-023-04723-2

**Published:** 2024-01-20

**Authors:** Sylvain Vanoli, Elisabeth Grobet-Jeandin, Olivier Windisch, Massimo Valerio, Daniel Benamran

**Affiliations:** Urology Department, Hôpitaux Universitaires de Genève, Rue Gabrielle-Perret-Gentil 4, 1205 Geneva, Switzerland

**Keywords:** Prostate biopsies, Virtual reality, Prostate cancer, Anxiety management, Local anesthesia

## Abstract

**Introduction and methods:**

Prostate biopsy (PB) is an essential step in the diagnosis and active surveillance of prostate cancer (PCa). Transperineal PB (TP-PB) is now the recommended approach and is mostly conducted under local anesthesia. However, this procedure can potentially cause anxiety for patients, given the oncological context and the fear of peri-procedural pain and complications. The objective of this narrative review is to summarize the currently available tools for the management of peri-interventional anxiety during TP-PB, with a particular emphasis on the potential role of virtual reality (VR) in this setting.

**Results:**

In TP-PB, preoperative anxiety can lead to increased pain perception, longer procedure time, and decreased patient satisfaction. Pharmacological and non-pharmacological approaches have been explored to reduce anxiety, such as premedication, deep sedation, education, relaxation techniques, hypnosis, and music therapy, albeit with mixed results. VR has recently emerged in the technological armamentarium for managing pain and anxiety, and the efficiency of this technology has been evaluated in various medical fields, including pediatrics, gastroenterology, urology, gynecology, and psychiatry.

**Conclusion:**

Despite the paucity of available data, VR appears to be a safe and effective technique in reducing anxiety in many procedures, even in frail patients. No studies have evaluated the role of VR in TP-PB. Future research should thus explore the optimal way to implement VR technology and any potential benefits for TP-PB patients.

## Introduction

Prostate cancer (PCa) is the second most commonly diagnosed cancer in men, with 1.4 million diagnoses worldwide in 2020 [1]. The screening process of patients at risk is based on digital rectal examination and PSA-level assessment, followed by prostate MRI in the subjects who test positive [2]. Despite the evolution of non-invasive technologies to predict the likelihood of PCa, prostate biopsies (PB) remain the essential diagnostic tool for definitive diagnosis. In the last decades, this procedure has evolved considerably due to advancements in medical technologies, with more than a million PB procedures currently performed every year in the United States alone [3]. PB can be performed via a transrectal (TR) or a transperineal (TP) approach [2]. The latter TP approach is at present recommended by learned societies to decrease infectious complications. As it is well tolerated and safe, it can be carried out under local anesthesia in an outpatient setting [4]. However, managing anxiety in patients who undergo PB under local anesthesia (LA) remains challenging, in part due to the fear over a potential oncological diagnosis, and in part because this procedure can cause pain or post-operative complications in the urogenital sphere [5].

Several interesting tools, such as hypnotherapy [6], nitrous oxide [7] or cognitive behavioral techniques [8], have been developed and used to improve the overall experience and comfort of patients undergoing PB. Among recently developed technologies, virtual reality(VR) has emerged as an efficient immersive tool. The capacity of this technology to divert attention has interesting applications in the medical field. VR appears to be a promising choice for lowering anxiety during many procedures. The purpose of this study is to review the management of peri-interventional anxiety during TP-PB and explore the potential role of VR.

## Methods

A review of the literature was performed using the National Library of Medicine database (https://www.ncbi.nlm.nih.gov). A Medline search was performed with a special emphasis on PB and VR-assisted medical procedures using combinations of the following (MeSH) terms: prostate cancer, prostate biopsy, transperineal biopsy, local anesthesia, pain, anxiety, virtual reality, and health-related quality of life. Additional articles were selected by cross-referencing the bibliography of previously selected articles. Articles published between 2000 and 2022 were considered. Older studies were selectively included only if historically relevant or in the case of lack of data in more recent publications. We identified a total of 91 relevant articles related to PB and VR, and after careful assessment, 26 of these articles were considered suitable for inclusion. Around 10 of them were focusing on anxiety management during PB and 15 on the use of VR for anxiety management in various medical fields.

## Results

### Transperineal prostate biopsy

The first PB procedures can be traced back to the beginning of the twentieth century when these were mainly performed extemporaneously before open TP prostatectomy under general anesthesia [3]. The introduction of transrectal ultrasound(TRUS) imaging revolutionized PCa detection by providing guidance for needle biopsy [3], helping to develop the concept of targeted biopsies for PCa. In the 1980s, TR biopsies under TRUS guidance became the gold standard for PCa detection [9]. The recent advent of prostate MRI and MRI to TRUS fusion targeted biopsies has further refined the diagnostic accuracy of PCa [10], allowing to personalize active treatment and to expand the concept of active surveillance for low-risk tumors [2].

Currently, both TR and TP approaches are still being used, even though the trend is shifting towards TP-PB [11]. Indeed, TP-PB has shown increased detection rate of anterior zone cancer and significantly lower risk of infectious complications [12]. The European Association of Urology guidelines already recommend the use of a TP approach if feasible [2], and a gradual shift to TP-PB is ongoing across the board.

Ensuring patient comfort throughout the procedure has always been a priority when establishing PB strategies. However, the TP approach caused more pain due to the additional layers of tissues and structures encountered to reach the gland [3], so it was traditionally performed under general anesthesia. The introduction of ultrasonographic tools changed this by enabling a more effective strategy for targeted LA [13]. Nowadays, LA starts with infiltration of perineal skin in the area of the needle entry points, followed by targeted periprostatic, subcutaneous and/or pudendal nerve block [14]. The most commonly used anesthetic is Lidocaine 1–2%, which can be buffered with sodium bicarbonate to minimize the burning sensation during injection [15]. On an oncological level, there is no difference in PCa detection or complication rates in PB under LA compared to sedation or general anesthesia [16]. This has allowed TP-PB to be performed in an ambulatory setting, with patients reporting only mild levels of discomfort. This eventually frees up valuable theater time and recovery resources that can be allocated to other surgery and limit the costs of the intervention [17]. A study evaluating the tolerability of TP-PB under LA on 48 men reported a score on the visual analogue scale (VAS) of pain of 5/10 during infiltration, 3/10 during US probe insertion, and 2/10 during biopsies [4]. Similarly, another study involving 1287 patients undergoing TP-PB showed similar results, highlighting that anesthesia infiltration was perceived as more painful than the biopsy procedure itself [18].

### Prostate biopsy and anxiety

Even though robust data on psychological impact of PB are scarce, qualitative and quantitative research suggest that they can be a source of anxiety for patients. In 2001, Zisman reported that 64% of patients undergoing TRUS-guided transrectal biopsies reported preoperative anxiety, with as high as 19% reporting severe anxiety requiring anxiolytic medications. The anxiety began at the moment the procedure was planned and peaked before results disclosure [19]. A more recent prospective study, possibly more representative of current practice showed that up to 49% of patients experience significant psychological distress related to the procedure, mainly caused by a fear of the procedure itself as well as its possible implications [5]. Another study analyzing the experience of patients with negative prostate biopsy showed that around 20% of them reported high level of tension-anxiety and psychological distress at the time of PB [20]. Many studies showed that higher anxiety level led to increased pain perception during the procedure, longer procedure times, and result in lower patient satisfaction [19, 21, 22]. Thus, identifying effective techniques to manage anxiety is essential for improving the patient experience. At diagnosis of PCa, clinical or subclinical anxiety and depression can be found in 30,3% and 20,3% of patients, respectively [23]. Post-biopsy symptoms, such as sepsis, hematuria or hemospermia, can also significantly increase anxiety levels [24]. Preoperative education and anxiety management can significantly reduce post-operative anxiety levels as well as post-operative complications [25].

Several tools have been developed to evaluate peri-procedural anxiety levels in patients, with most of them consisting of validated self-evaluation scores. The Hamilton Anxiety Rating Scale(HAM-A), dating back to 1959 and translated to various languages, is a 14-item score that is still used in clinical practice [26]. Of note, it has been criticized for its limited ability to distinguish between anxiolytic and antidepressant effects [26]. The State-Trait anxiety inventory(STAI) is currently the most commonly used questionnaire and consists of two separate subscales. The first is the S-form, which evaluates the current state of anxiety and can be easily repeated at different times of a procedure. The second is the T-form, which evaluates a patient’s general tendency towards anxiety. The STAI consists of a total of 40 short questions(20 for each subscale) with answers from 1 to 4, and provides an indication of the level of anxiety at a given moment and the overall propensity for anxiety [27]. Other validated tools, such as the Beck Anxiety Inventory(BAI) or Hospital Anxiety and Depression Scale-Anxiety(HADS-A), are also often used. Finally, the Amsterdam Preoperative Anxiety and Information Scale(APAIS) [28] and the Surgical Fear Questionnaire(SFQ) [29] are both validated tools that enable evaluation of preoperative anxiety levels.

All these questionnaires are subject to being influenced by the patients’ subjectivity. To obtain objective measurements, vital signs are commonly used during procedures to detect any physiological autonomic response to anxiety, which may manifest as increased tension and/ or heart rate[30].

### Reduction of anxiety in PB

To counter potential adverse effects of anxiety, various approaches have been explored. One approach includes the use of pharmacological agents. For instance, the use of premedication like benzodiazepines or sedatives, to promote relaxation and alleviate anxiety, has been investigated. A randomized controlled trial (RCT) conducted in 2014 that included 60 patients undergoing TR-PB found no improvement on pain perception after administration of diazepam as premedication [31]. Another pharmacological approach involves deep sedation and seems to be a more efficient option for PB. In 2020, a RCT that included a total of 135 patients undergoing TR-PB found that, compared to LA alone, deep sedation significantly decreased anxiety and pain scores (p < 0.001) [32]. Deep sedation, however, has logistic and resources implications making this option less suitable for such a common procedure.

This has naturally prompted researchers to explore non-pharmacological options to manage anxiety in PB. A simple approach that focuses on education and accompaniment of the patient has been proposed. This includes an explanation of the causes of anxiety, its cognitive, physical and behavioral symptoms, as well as the treatments and techniques used to reduce anxiety [25]. This empowerment of patients could be achieved using video-based education and seems to be effective in significantly reducing anxiety levels. Indeed, in a RCT conducted in 2014, Tarhan et al. [33] evaluated 123 patients who were invited to watch an instructional video accompanied by a specialized nurse, reporting a reduced STAI-S Form score of 35.7 compared to 42.3 in the control group (p = 0.01). Other behavioral methods include relaxation and breathing techniques, investigated in a RCT by Grinberg et al. [8] in 2019. A total of 20 patients received a single 15-min psychoeducation and diaphragmatic breathing session and were also reminded to practice the diaphragmatic breathing during PB. This approach resulted in a significant decrease in heart rate and STAI-S scores.

Hypnosis is another common tool for preoperative anxiety management. Its application in urology has shown interesting results on anxiety and pain during rigid cystoscopy [34], as well as in TR-PB. A RCT in 2014 conducted by Hizli et al. [6] found that hypnotherapy sessions using a 10-min preoperative session could significantly decrease anxiety scores. Another approach utilizes nitrous oxide(N_2_0), a short-action inhalation anesthetic commonly used in pediatrics and emergency in an outpatient setting. Its use has been proven safe and efficient with minimal side effects and rapid clearance [35]. However, a RCT in which patients received N_2_0 3 min prior to biopsies failed to show a significant reduction in pain and anxiety levels [7]. Finally, the use of music on anxiety has been evaluated during various surgical and endoscopic procedures [36]. Its use in a RCT showed an autonomous response with a decrease in post-biopsy blood pressure, with no significant reduction of anxiety scores [37].

The techniques mentioned above are still used marginally and their effectiveness needs further confirmation before wider use. Of note, no studies have evaluated the role of these techniques in reducing anxiety during TP-PB.

The above-mentioned studies are summarized in Table 1.

**Table 1:** Studies evaluating the effect of non-pharmaceutical tools for anxiety reduction in prostate biopsy

Study	Year	Type	Intervention	Pb	Outcomes	Patients	Tools	Results
Grinberg et al. [8]	2019	RCT	Diaphragmatic breathing	TR	Anxiety	40 (20:20)	STAI-S Vital signs	Significantly decrease of anxiety from pre- to post-procedure. Significant decrease in heart rate with diaphragmatic breathing
Hizli et al. [6]	2014	RCT	Hypnotherapy	TR	Anxiety Pain	64 (32:32)	HAS BAI VAS (pain)	Significant decrease pain and anxiety scores with hypnotherapy
Spie et al. [7]	2008	RCT	Nitrous oxide	TR	Anxiety Pain	102 (nitrous oxide *n* = 55: control *n* = 47)	STAI-S VAS (pain)	No significant difference in anxiety scores, non-significant decrease of pain intensity with NO
Tarhan et al. [33]	2014	RCT	Video-based education	TR	Anxiety Pain	246 (123:123)	STAI-S STAI-T VAS (pain)	Significant decrease of anxiety post-information. No difference in pain
Tsivian et al. [37]	2011	RCT	Music or noise-cancelling headphones	TR	Anxiety Pain	88 (music *n* = 31, headphones *n* = 29, control *n* = 28)	STAI-S STAI-T VAS Vital signs	No significant difference in anxiety scores between groups. Post-biopsy diastolic blood pressure increased in the control and headphones groups but remained stable in the music group

*PB* prostate biopsies, *TR* transrectal, *RCT* randomized controlled trial, *STAI* State-Trait Anxiety Inventory, *STAI-S* form state, *STAI-T* form trait, *VAS* Visual Analog Scale, *HAS* Hamilton Anxiety Scale, *BAI* Beck Anxiety Scale

### Virtual reality

As new advances in technology slowly push the world into an era of VR, the field of medicine is already seeing some of its application being implemented in clinical practice. Augmented reality(AR) and VR have the potential of transforming and improving healthcare in a way that still needs to be defined [38]. Numerous devices that incorporate AR or VR have been reviewed and authorized for marketing by the U.S Food and Drugs Administration (FDA) in the United States and this trend is expected to continue in the near future.

AR is defined as the superimposition of digital elements onto real-world elements seen through a camera or a display [39]. Its main goal is to provide intra-operative guidance by overlaying preoperative imaging to the operation field. Its development is currently underway and is already being used for training and preoperative planning [40]. In urology, AR has been used in a number of scenarios. For instance, during a simulation of ureteroscopy, AR has allowed a significant improvement in operating time(absolute difference, −73 s; 95% CI −115 to −30; *p* = 0.0011) and performance(OSAT scores, absolute difference, 4.1 points; 95% CI 2.9–5.3; *p* < 0.0001) [41]. Another example in urology is a transurethral bladder tumor resection (TURBT) simulator with AR, which showed significant differences in procedure time (*p* = 0.007), resectoscope movement (*p* = 0.005), and accidental bladder injury (*p* = 0.003) after training [42]. This technology is mainly intended for doctors and technicians with the aim of improving the state of science and the quality of interventions.

**Fig. 1:**
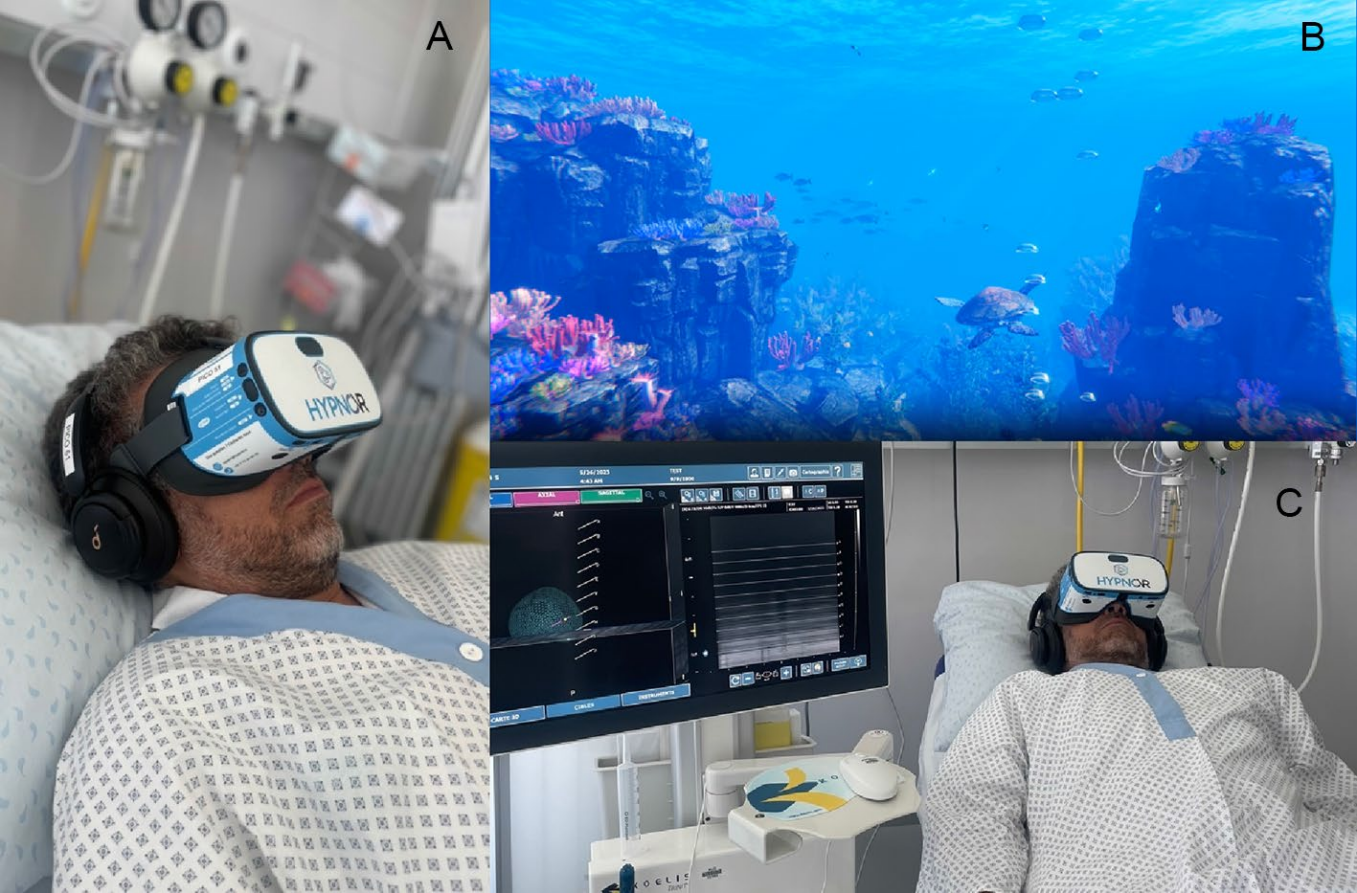
TP-PB with VR assistance in an outpatient setting. **A, C** A simulated patient equipped with the VR headset (personal picture from the authors). **B** An example of one of the immersive computer-generated worlds provided by the HypnoVR^©^ (HypnoVR, Strasbourg, France) device

VR is a visual and auditory immersion of the patient in a computer-generated artificial environment. It is achieved with a VR headset and earphones, which trick patient’s senses into thinking in a different three-dimensional world, as illustrated on Fig. 1. Originally developed for commercial purposes, the potential application of this technology to medicine was recognized promptly. Since its commercialization in the last 5 years, medical practitioners are witnessing an increasing use of VR in various fields, such as rehabilitation, mental health, pain management, neurological or pediatric care, with encouraging results. A rehabilitation program of patients with Parkinson’s disease showed greater improvement in balance and gait for patients treated with VR rehabilitation compared to conventional physiotherapy [43]. Treatment of post-traumatic stress disorder symptoms and phobias has shown robust reductions through exposure to VR interventions [44]. Two meta-analyses evaluated the role of VR use in patients with other mental disorders such as anxiety, depression, schizophrenia, and phobias, supporting that VR could successfully decrease the severity of symptoms, underlining, however, that VR therapies were not mature enough yet for clinical application [45, 46]. Of note, VR has shown some limitations, particularly in patients that display symptoms of motion sickness like nausea, fatigue, and headaches or dry eyes due to overheating of the device [47].

### VR and anxiety reduction

A short communication focusing on the impact of VR on anxiety and pain during TP-PB was recently presented [48]. They included 60 men and assessed pain and anxiety with a Visual Analog Scale. Pre-procedure anxiety levels were similar in both groups (VR 5.8 vs Control 5.2, *p* = 0.18). Peri-procedure anxiety level (VR 4.1 vs Control 6.9, *p* < 0.01) and pain (VR 4.0 vs Control 5.6, *p* < 0.01) were significantly reduced. A published RCT that included 96 patients undergoing TR-PB, VR was shown to significantly reduce pain, diastolic pressure, pulse, and respiratory rate (*p* < 0.05) [49]. Another group of urologists was intrigued by the role of VR for reduction of pain and anxiety in patients undergoing vasectomy in an outpatient setting. Surprisingly, VR did not reduce pain in comparison with the control group, and higher levels of anxiety were reported in the group of patients using VR [50]. The authors of this study suggested that the quality of the VR device was either too poor or did not match with the patients’ interests.

Various groups of gynecologists also showed interest in the role of VR for anxiety reduction. As an example, in a RCT conducted in 2018 that included 80 women diagnosed with breast cancer, VR and morphine combined showed a significant reduction in pain and anxiety when compared to morphine alone (*p* < 0.001) [51]. In a pilot study from 2020 that included 30 women undergoing first-trimester surgical termination of pregnancy, VR-induced relaxation and distraction helped to reduce anxiety in patients during the entire procedure (*p* < 0.01) [52]. Furthermore, in a meta-analysis including 446 women from 8 RCTs, VR was shown to be effective in reducing anxiety (*p* = 0.03), increasing satisfaction (*p* = 0.004), and improving pain management (*p* < 0.001) during normal labor [53].

Pediatrics have been pioneers in the use of VR in the medical field, due to their particular population of patients. The non-invasiveness and absence of any pharmaceutical substance of VR has been this technology particularly interesting for use with children, whose anxiety management can be challenging. In 2019, Eijlers et al. evaluated the effectiveness of VR to reduce anxiety in a RCT that included a total of 200 children aged 4–12 years undergoing day care surgery under general anesthesia [54]. VR was administered before surgery and had no beneficial effect on anxiety or pain. However, following a more painful surgery, children in the VR group needed rescue analgesia significantly less often (*p* = 0.002). In 2021, a total of 50 children aged 6–12-years old were included in a RCT evaluating the effect of VR compared to standard screen TV in reducing anxiety for buccal infiltration anesthesia. No significant difference was observed between the groups, but female and younger patients showed higher pain scores during the dentistry procedure [55]. Two recent meta-analyses that included a maximum of 17 studies evaluating the effect of VR on pain and anxiety in a pediatric population concluded that VR is an effective distraction intervention to reduce pain and anxiety in children [56, 57].

Finally, other medical fields have also explored the role of VR in anxiety reduction. In gastroenterology, VR has been used prior to endoscopic procedures to reduce anxiety and has shown promising results, reducing anxiety significantly in patients with a higher anxiety level (STAI-score ≥ 45) at baseline (*p* = 0.007) [58]. Another group evaluated the effect of VR in a RCT of 60 patients undergoing colonoscopy, showing that VR significantly reduced pain (*p* < 0.03) and anxiety (*p* < 0.001) during the procedure [59]. In a RCT of 40 patients diagnosed with chronic venous disease and treated by intravenous radiofrequency ablation, VR significantly decreased procedural anxiety (*p* < 0.01)[60]. Similar results were found in a study on the use of VR for bronchoscopy [61]. One meta-analysis evaluated the effectiveness of VR in reducing pain, fear, and anxiety in needle-related procedures in young patients and found a significant reduction of pain but no effect on fear and anxiety [62]. A study on patients receiving chemotherapy did not show significant decrease of anxiety compared to control group or a biophilic environment [63].

A summary of the main results of these studies can be found in Table 2.

**Table 2:** Studies evaluating the role of VR for anxiety reduction in various medical interventions and conditions

Study	Year	Type	Intervention/condition	Outcomes	Patients	Tools	Results
Baradwan et al. [53]	2022	Meta-analysis	Normal labor	Pain, anxiety, satisfaction	446	VAS (pain)	Significant reduction of pain score, anxiety, and satisfaction with VR
Brewer et al. [60]	2021	Prospective, RCT	Saphenous vein radiofrequency ablation	Pain, anxiety	40 (VR 20; control 20)	WBS VAS (anxiety)	No significant reduction of pain; significant decrease of anxiety during the procedure with VR
Czech et al. [62]	2021	Meta-analysis	Needle-related procedures	Pain, anxiety, fear	–	WBS, FPS	Significant decrease of pain with VR. No significant decrease of fear and anxiety with VR
Dings et al. [48]	2021	Prospective, non-RCT	Vasectomy	Pain, anxiety	141 (VR 37; 2D-VG 43, control 61)	VAS (pain) STAI-AD	No significant difference in pain and stress with VR. Higher levels of anxiety found in the VR group
Eijlers et al. [54]	2019	Prospective, RCT	Day care surgery under general anesthesia in children	Pain, anxiety, child behaviour, parents’ anxiety	200 (VR 100; control 100)	mYPAS, FPS, CBCL, STAI	No significant reduction of pain, child and parents’ anxiety or emergence delirium with VR
Eijlers et al. [56]	2018	Meta-analysis	Children undergoing medical procedures	Pain, anxiety	859	–	Reduction of pain and anxiety with VR
Felemban et al. [55]	2021	Prospective, RCT	Buccal infiltration anesthesia in children	Pain, anxiety	50 (VR 25; control 25)	WBS monitoring	No significant difference between VR and control group. Female and younger patients show higher pain scores during procedure
Genç et al. [47]	2022	Prospective, RCT	Transrectal prostate biopsy	Pain, vital signs	96 (VR 32; SB 32, control 32)	VAS (pain) monitoring	Significant decrease of pain, diastolic pressure, pulse and respiratory rate in the VR group
Ioannou et al. [46]	2020	Systematic review	Mental disorders	Pain, anxiety, fatigue, depression	–		Distraction by VR is a potential effective symptom management mechanism in mental disorders
Karaveli et al. [67]	2021	Prospective, RCT	Colonoscopy	Pain, anxiety, satisfaction	60 (VR 30; control 30)	VAS (pain) STAI-S	Significant reduction of pain and anxiety with VR
Kim et al. [58]	2023	Prospective, non-RCT	G-E endoscopy	Pain, anxiety, satisfaction	40 (VR 20; control 20)	VAS (pain) STAI-S	Significant reduction anxiety in patients with higher baseline anxiety level using VR
Lachkar et al. [61]	2022	Prospective non-RCT	Bronchoscopy	Anxiety	20	VAS (anxiety)	Reduced anxiety
Mohammad et al. [51]	2018	Prospective, RCT	Breast cancer	Pain, anxiety	80 (VR 40; control 40)	VAS (pain) STAI-S	Significant reduction of pain, and anxiety with VR
Simonetti et al. [57]	2022	Meta-analysis	Children undergoing surgery	Anxiety	602	mYPAS, YPAS	Effectiveness of VR in reducing anxiety
Sridhar et al. [52]	2020	Prospective, non-RCT	1st-trimester surgical termination of pregnancy	Anxiety	30 (VR 15; control 15)	VAS (anxiety) APAIS	Significant reduction anxiety in some patients using VR
Verzwyvelt et al. [63]	2021	Prospective non-RCT	Chemotherapy	Pain, anxiety, vital signs, saliva cortisol	33	VAS (pain, anxiety)	No significant difference in vital signs, cortisol, pain or distress in a control, VR or biophilic environment
Wiebe et al. [45]	2021	Systematic review	Mental disorders	Diagnostic and treatment of mental disorders	–	–	VR is not yet mature for clinical application in mental disorder diagnostic and treatment

–, not reported, *VR* virtual reality, *SB* squeeze ball, *G-E* gastroenterology, *VAS* Visual Analog Scale, *2D-VG* 2-dimensions video glasses, *STAI-AD* State-Trait Anxiety Inventory for Adults, *APAIS* Amsterdam Preoperative Anxiety and Information Scale, *WBS* Wong–Baker Scale, *mYPAS* Modified Yale Preoperative Anxiety Scale, *FPS* Faces Pain Scale, *CBCL* Child Behaviour Checklist, *YPAS* Yale Preoperative Anxiety Scale.

## Discussion

PB is a stressful procedure for patients and can be source of anxiety, resulting in a negative experience. Since pharmaceuticals approaches may have significant side effects, many non-pharmaceutical tools have been evaluated to improve the tolerability of the procedure. Techniques such as video-education, diaphragmatic breathing or hypnotherapy have proven useful to some degree in reducing anxiety, whereas other techniques like N_2_0 and musical therapy have shown limited improvement. More recently, VR emerged as a new and promising option for management of anxiety in various medical procedures.

The evaluation of anxiety can be difficult due to its subjective nature and the fact that it is influenced by multiples factors. Although validated questionnaires have proven to be the most reliable tool and are mandatory for anxiety assessment, they are still subject to limitations like variability between patients and difficulty to discriminate between symptoms of anxiety and depression [64]. This has led to a scarcity of data on anxiety management in urology. Further investigation is needed to define the best tools for better management of anxiety. Indeed, paving the way to personalized medicine, improvement in quality of life and comfort of patients have become the cornerstone of patient care, especially in case of a new oncological diagnosis. In the case of PCa, TP-PB is usually the first invasive procedure in the oncological path of the patient. A better experience for the patient will probably increase compliance and willingness to undergo further biopsy as these might be indicated in the era of tissue-preserving approaches. Optimal management of anxiety allows for a better overall experience both for the patient and the urologist. Indeed, we can suppose that an anxious and agitated patient during TP-PB might compromise the procedure and, therefore, the accuracy of the oncological diagnosis. It could potentially also expose the patient to a prolonged operating time or an increase in post-operative complications.

The emergence of VR opens many opportunities for its application in the medical field. It seems to be an appropriate tool for anxiety management, and appears to be safe, well tolerated and effective in various medical settings. Given that it was first developed for commercial use, the price of VR technology remains affordable compared to other medical devices. Democratization of VR may eventually reduce its costs further, making it a cost-effective tool for anxiety management in PB, especially knowing it can minimize the need for sedation anesthesia or general anesthesia. Like other non-pharmaceutical tools, the extension of VR use to other outpatient interventions, such as cystoscopy or shock-wave lithotripsy (SWL), can be easily foreseen. At first, introducing these new devices in a medical setting to patients not accustomed to this technology may remain a challenge. Thorough explanation and accompaniment are further needed to maximize the benefits and compliance of this technology. Also, reluctance to VR in the medical field might soon decrease through the generalization of the use of virtual technologies in daily life. In the future, these devices could also be combined with other existing management like hypnosis. A recent review of the literature, however, underline that we cannot affirm the added value at the moment and further studies will be necessary [65].

The aim of the present review was to give a detailed description of the possibilities to better manage anxiety during PB and especially TP-PB, with a focus on VR. It is important to acknowledge several limitations. First, the study primarily relies on a narrative review of the existing literature, which introduces the potential for selection bias as the choice of articles included in the review is subjective. The study includes a wide range of medical procedures beyond TP-PB to discuss the application of VR, and these findings may not be directly translatable to the specific context of TP-PB. Also, the quality of the VR technology used in different studies may vary, which can impact the outcomes. While this review underscores the potential of VR as a tool for managing anxiety during TP-PB, these limitations warrant caution in generalizing the findings of VR to the TP-PB context, and further research is needed to validate the effectiveness of VR in reducing anxiety in this specific medical procedure. Preoperative and postoperative anxiety should be further assessed by validated questionnaires. A multicentric European RCT including interventions associated with VR in different surgical fields started recruiting and are expected to publish results this year [66]. These broader studies are expected to underline the value of VR in surgery. Further results on this topic are eagerly awaited.

## Conclusion

From open TP-PB under general anesthesia to fusion-targeted PB under LA with VR assistance, recent technological developments seemed to have revolutionized TP-PB. In addition to optimal oncological care, the comfort and serenity of patients should always be a priority for the surgeon. Among many tools recently evaluated for anxiety management in various medical fields, VR seems to be an effective method to reduce anxiety. Its application in TR-PB should be encouraged and RCT studies that evaluate its effects on anxiety are eagerly awaited.

## Data Availability

Data that support the findings of this study are available upon reasonable request.
